# Gut Microbiota: The Brain Peacekeeper

**DOI:** 10.3389/fmicb.2016.00345

**Published:** 2016-03-17

**Authors:** Chunlong Mu, Yuxiang Yang, Weiyun Zhu

**Affiliations:** Jiangsu Key Laboratory of Gastrointestinal Nutrition and Animal Health, Laboratory of Gastrointestinal Microbiology, College of Animal Science and Technology, Nanjing Agricultural UniversityNanjing, China

**Keywords:** gut microbiota, nervous system, gut–brain axis, anxiety, circadian rhythm, pattern recognition receptors

## Abstract

Gut microbiota regulates intestinal and extraintestinal homeostasis. Accumulating evidence suggests that the gut microbiota may also regulate brain function and behavior. Results from animal models indicate that disturbances in the composition and functionality of some microbiota members are associated with neurophysiological disorders, strengthening the idea of a microbiota–gut–brain axis and the role of microbiota as a “peacekeeper” in the brain health. Here, we review recent discoveries on the role of the gut microbiota in central nervous system-related diseases. We also discuss the emerging concept of the bidirectional regulation by the circadian rhythm and gut microbiota, and the potential role of the epigenetic regulation in neuronal cell function. Microbiome studies are also highlighted as crucial in the development of targeted therapies for neurodevelopmental disorders.

## Introduction

The collective genome of the gut microbiota is estimated to contain many more genes than the human genome (Xu and Gordon, 2003). Gut microbiota is functionally diverse and participates in carbohydrate metabolism, fiber degradation, and immune maintenance. In addition, gut microbiota has also been implicated in regulating neurophysiological-governed behaviors, such as stress, autism, pain, and multiple sclerosis (Cryan and Dinan, 2012). Gut microbiota is found to regulate the neurophysiological behaviors through immune, endocrine and neural pathways (Collins et al., 2012). It is now clear that the gut–brain communication is bidirectional. On one hand, changes in the microbial community affect behavior. On the other hand, perturbations in behavior alter the composition of the gut microbiota (Collins and Bercik, 2009). However, the microbial community is affected by many environmental factors and host-related factors (physiological status; Lozupone et al., 2012). Since changes in the composition of the gut microbiota are associated with the behavioral and cognitive alterations (Cryan and Dinan, 2012), a healthy microbiota community is essential for a normal regulation of the microbiota–gut–brain axis. Among the potential factors regulating the axis, microbial metabolites may be the major mediators (Cryan and Dinan, 2012). In this review, we discuss recent studies on the microbial regulation of the brain health and the potential of the host-microbiota interaction in regulating various neurophysiological behaviors, highlighting the role of the gut microbiota as a “peacekeeper” in regulating the brain-controlled function and behavior.

## Gut Microbiota and Factors that Drive Variations in Microbiota Composition

The gut contains more than 1,000 bacterial species, as being identified by culture-independent approaches (Rajilic-Stojanovic and de Vos, 2014). Firmicutes and Bacteroidetes are the predominant phyla (Collins et al., 2012). The distribution of the gut microbiota shows the spatial and temporal variation in both humans (Eckburg et al., 2005; Yatsunenko et al., 2012) and rodents (Gu et al., 2013; Maurice et al., 2015). Nonetheless, different microbes with metabolic and/or immunological regulation abilities colonize the gut, generating a complex interaction network within the microbes or between the gut microbiota and the host. The complexity of the microbial community, together with its diversity, stability, and resilience, enables the gut microbiota to adapt readily to the gut environment (Lozupone et al., 2012). A typical mutualism interaction is the degradation of fiber in the gut. Fiber degradation occurs through a mutualism interaction with the host, whose digestion system itself does not have this function (Velasquez-Manoff, 2015). To complement the deficiency, intestinal microbes use glycoside hydrolases and polysaccharide lyases to degrade the fiber into short-chain fatty acids while these acids benefit the host (El Kaoutari et al., 2013). The resilience ability is also an important property of the gut microbiota. The ability of certain microbiota members which dephosphorylate lipopolysaccharide (LPS) is important for the microbiota resilience during inflammation-induced disturbance (Cullen et al., 2015).

Recent advances in “omics” have expanded our knowledge of the multitude functions of the gut microbiota. Within the gut microbiota, some members, such as *Lactobacillus* and *Bifidobacterium* species, are widely used as probiotics to promote intestinal homeostasis (Bron et al., 2012). Others, such as *Akkermansia muciniphila* (Derrien et al., 2008) and *Bacteroides thetaiotaomicron* (Marcobal et al., 2011), are well-known for their role in the mucin degradation. Clearly, the gut microbiota adapts well in the gut with varying functions. Identification and clarification of these functions provide the basis for manipulating microbiota in order to maintain homeostasis and contribute to setting the targets for developing the therapy against disorders.

**FIGURE 1:**
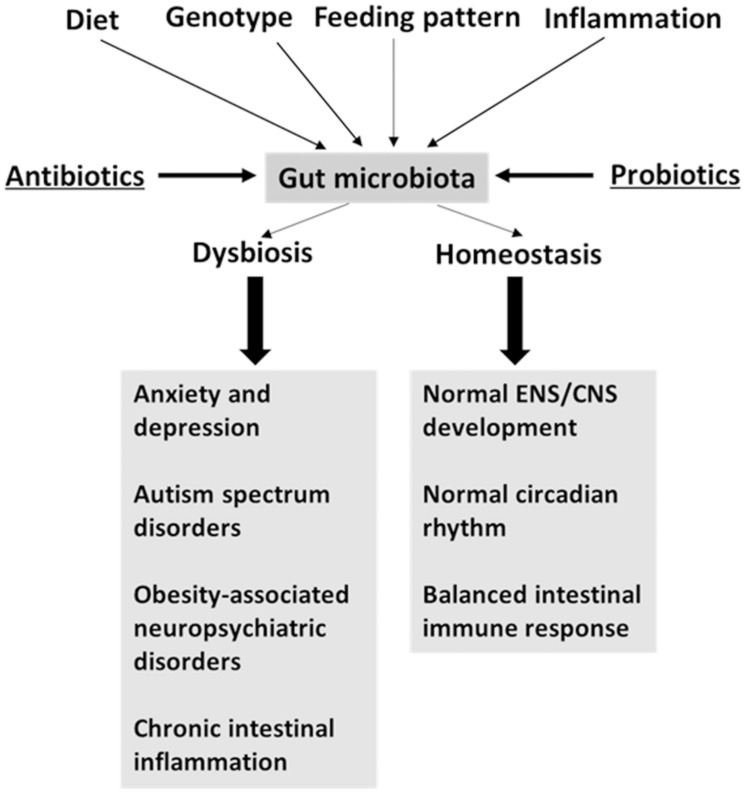
**Factors driving the variation of gut microbiota may affect brain function.** Antibiotics and probiotics treatment are microbiota-targeted interventions.

Various factors, such as genotype, diet, inflammation, and time of feeding, affect the microbiota community (**Figure 1**), as discussed below. Take the genotype as an example, the inbred mice with different genetic backgrounds own different composition of the gut microbiota in the cecal lumen (Campbell et al., 2012). Diet is known to affect the composition of the gut microbiota. We find that a high-protein diet alters the colonic microbiota in rats (Mu et al., 2016). In pig models, the pig breed (Yang et al., 2014a) and dietary differences in the amount of starch (Sun et al., 2015) affect the composition of the gut microbiota. Other studies which use a pig model, the composition of the gut microbiota from different sites, such as the lumen and epithelial wall, varies, as well as their ability to utilize amino acids (Dai et al., 2013; Yang et al., 2014b). Pathogen infection also changes the microbial community. *Citrobacter rodentium* infection increases the abundance of Enterobacteriaceae in the colon of mice (Lupp et al., 2007). A recent research shows that feeding patterns alter the daily cyclical composition of the gut microbiota in mice (Zarrinpar et al., 2014). These facts indicate that multiple variables affect the composition of the gut microbiota. Additionally, the aforementioned factors can also affect the intestinal function, enteric nervous system (ENS) function, and central nervous system (CNS) function.

## The Gut Microbiota is Essential for Enteric and Central Nervous System Development

The primary evidence of the gut microbiota as a brain peacekeeper is the discovery that gut microbiota regulates nervous system development. Indeed, gut microbiota regulates postnatal and adult development of the ENS in rats and mice (Dupont et al., 1965; Neufeld et al., 2013; Collins et al., 2014). The ENS controls intestinal motility and signals to the CNS. The myenteric plexus of the jejunum and ileum of germ free (GF) mice is abnormal compared to that of specific pathogen-free (SPF) mice. The abnormality is related to a decrease in the nerve density and the number of neuronal cell bodies per ganglion, while an increase in nitrergic neurons on postnatal day 3 (Collins et al., 2014). In addition, gut microbiota may also affect enteric glial cells. Enteric glial cells are essential components of the ENS and act as a link in the gut–brain axis (Collins et al., 2012). The gut microbiota in the ileum is able to regulate both the initial colonization and homeostatic flow of glial cells in the intestinal mucosa of mice (Kabouridis et al., 2015). In GF mice, the average number and density of mucosal enteric glial cells are significantly reduced compared to that of normal mice (Kabouridis et al., 2015). This finding suggests that microbiota and microbial products may potentially affect gastrointestinal homeostasis via enteric glial cells. Furthermore, enteric glial cells, at least in the ileum, link microbial cues with the host’s nervous system. However, further research is required to determine the nature of the relationship between enteric glial cells and abnormal intestinal diseases or neuropsychiatric disorders.

Gut microbiota can also regulate the survival of enteric neurons and the gastrointestinal motility, possibly through its recognition by toll-like receptors. The depletion of the gut microbiota by antibiotics leads to ENS anomalies and at the same time decreases the expression of glial cell line-derived neurotrophic factor (GDNF; Brun et al., 2013). An another study shows that GF mice, wild-type mice depleted of the gut microbiota, and *Tlr4*-deficient mice have delayed gastrointestinal motility and reduced numbers of nitrergic neurons (Anitha et al., 2012). However, LPS treatment promotes the survival of enteric neurons through the TLR4-nuclear factor-kappa B pathway (Anitha et al., 2012). Therefore, the TLR4-mediated interaction between enteric neurons and microbiota may be important for the function of the ENS.

It is worth noting that gut microbiota also regulates the permeability of the blood–brain barrier in mice. The GF mice have a lower expression of occludin and claudin-5 in the frontal cortex, striatum, and hippocampus than SPF mice (Braniste et al., 2014). The integrity of tight junctions is essential for maintaining the blood–brain barrier function. In GF mice, monocolonization by *Clostridium tyrobutyricum* or *Bacteroides thetaiotaomicron*, and sodium butyrate treatment in mice decrease the permeability of the blood–brain barrier as compared to that of control GF mice by up-regulating tight junction proteins (Braniste et al., 2014). These results suggest that the gut microbiota or microbial products may be essential for establishing normal blood–brain barrier permeability.

## Gut Microbiota is Involved in Behavioral Regulation

The GF model provides a direct way to study how gut microbiota regulates behavior (Cryan and O’Mahony, 2011). In GF rodents, the expression of the hypothalamic corticotrophin-releasing factor (CRF) gene is up-regulated, while the concentration of circulating corticosterone is increased after acute stress, as compared to that of SPF rodents (Sudo et al., 2004; Clarke et al., 2013; Crumeyrolle-Arias et al., 2014). These changes lead to elevated activity of the hypothalamic–pituitary–adrenal axis. In practice, the GF rodent and their SPF counterparts are commonly used to compare the response to anxiety. However, the results show some discrepancies.

Previous studies have demonstrated the reduced anxiety in GF female Swiss mice (Neufeld et al., 2011; Clarke et al., 2013) and GF male NMRI mice (Heijtza et al., 2011) compared to SPF mice. On the contrary, other studies find the increased anxiety in GF male F344 rats (Crumeyrolle-Arias et al., 2014) and GF BALB/c mice (Nishino et al., 2013). Several factors, including the methodology and genetic backgrounds of the animals, have been proposed to explain the reported differences (Crumeyrolle-Arias et al., 2014). With regard to the genetic background, the F344 rat and BALB/c mice may be genetically prone to anxiety, whereas the NMRI and Swiss mice may be less prone to anxiety (Crumeyrolle-Arias et al., 2014). Collectively, these findings suggest that the gut microbiota affects the sensitivity of rodents to stress-induced anxiety. However, some of the aforementioned studies of GF and SPF rodents do not explore the composition of the gut microbiota of the SPF group. Studies have also failed to consider the effect of different strains and diets on the functional capacity of the gut microbiota and their metabolites.

As well as regulating the stress-related behavior, the gut microbiota also regulates appetite by affecting the production of gut hormones, indicating a paradigm of the microbiota–gut–brain axis. Appetite is modulated largely by the gut–brain axis, a core function that controls energy homeostasis by balancing energy intake and energy expenditure, so as to maintain energy reserves (Abizaid et al., 2006; Morton et al., 2006). It is known that gut hormones can regulate appetite (Murphy and Bloom, 2006). Gut hormones are produced by enteroendocrine cells, which exist along the intestinal mucosa from the stomach to the distal colon and account for about 1% of gut mucosal cells (Vrieze et al., 2010).

The gut hormones peptide tyrosine-tyrosine (PYY) and glucagon-like peptide 1 (GLP-1) are produced in gut L cells and exert anorectic functions (Murphy and Bloom, 2006). PYY and GLP-1 bind receptors in the nerve ends of ENS and transmit nutrient signals to the hypothalamo-brain stem network along the vagus nerve, thus regulating appetite (Rasoamanana et al., 2012). The microbial products from degradation and fermentation of dietary fiber, such as acetate, propionate, and butyrate, affect the production of PYY and GLP-1. For example, acetate and butyrate are sensed by the G protein-coupled receptors GPR41 and GPR43, which then induce PYY and GLP-1 (Cani et al., 2013). Colon-derived acetate induces the up-regulation of pro-opiomelanocortin and down-regulation of agouti-related peptides in the hypothalamus, which lead to appetite suppression (Frost et al., 2014). In healthy humans, colonic delivery of propionate increases the plasma level of PYY and GLP-1, and decreases appetite (Chambers et al., 2014). *In vitro*, propionate induces the production of PYY and GLP-1 from primary cultured human colonic cells (Chambers et al., 2014). Propionate feeding also activates intestinal gluconeogenesis gene expression via a gut–brain neural circuit, which is dependent on GPR41 signaling in rats (De Vadder et al., 2014). Although acetate, propionate, and butyrate can affect gut hormone, their direct impact on neural function is unclear. A study in cultured PC12 cells shows that propionate and butyrate treatment affect the expression of genes involved in catecholaminergic neurotransmission (Nankova et al., 2014).

In summary, the literatures suggest that both the microbiota community and microbial metabolites are involved in mediating the microbiota–gut–brain axis during appetite regulation.

## Gut Microbiota Manipulation Regulates Behavior and Cognitive Function

To understand the potential of microbiota manipulation in treating psychiatric disorders, it is important to know how the psychiatric disorders alter the composition of the gut microbiota. It is also essential to understand whether the gut microbiota is a causal factor for psychiatric disorders. If so, some key members of the gut microbiota that are affected by the psychiatric disorders may be of therapeutic importance for restoring a normal microbiota community and behaviors. These topics will be addressed as follows.

### Behavioral and Cognitive Alterations Affect Gut Microbiota

The changes in the composition of the gut microbiota during psychiatric disorders have been widely recognized. For example, the increase in *Lactobacillus* and decreases in *Prevotella* (Kang et al., 2013), and the decrease in *Bifidobacterium* (Finegold et al., 2010; Adams et al., 2011) are observed in children with autism spectrum disorders (ASDs). Specifically, an increase in fecal Enterobacteriaceae and *Alistipes*, and a decrease in fecal *Faecalibacterium* and *Ruminococcus* are observed in patients with major depressive disorder, which are accompanied by a low level of brain-derived neurotropic factor (BDNF) in the blood (Jiang et al., 2015). BDNF is a key neurotrophin involved in neuronal growth and survival (Cryan and Dinan, 2012).

Exposure to stress is widely reported to reduce the abundance of *Lactobacillus* species (Galley and Bailey, 2014), which raises the possibility of using probiotic *Lactobacillus* sp. to alleviate the stress response. Our previous study shows that weaning stress reduces the abundance of *Lactobacillus* sp. in the stomach, jejunum, and ileum, while increases the abundance of the pathogen *Streptococcus suis* in the stomach of piglets (Su et al., 2008b). Similar findings are also observed in the hindgut of piglets after weaning (Su et al., 2008a). A related question is the potential impact of maternal stress on the microbiota of newborns. In a study using a primate model, prenatal stress by acoustical startle in female monkeys leads to decreased numbers of bifidobacteria and lactobacilli in the feces of infant monkeys (Bailey et al., 2004). However, such results should be interpreted cautiously, due to the reason that the results about bacterial processes in the gut cannot be simply based on analyses of fecal bacteria. In rhesus macaque, the fecal bacterial community shows a strong correlation with that of the colonic lumen and mucosa and a moderate correlation with that of the distal small intestine (Yasuda et al., 2015). The microbiota at specific sites in the gut should be studied when examining gut bacterial processes in the gastrointestinal tract. These alterations in microbiota members under certain disease condition can provide an indicator for a clinical diagnosis.

Beyond the impact on the microbial communities, psychiatric comorbidity may also affect colonic functions. In a mouse model of depression using olfactory bulbectomy female C57BL/6 mice, changes in neural behavioral increase colonic muscle contractility and tissue serotonin concentration and alter the fecal microbiota profile, but have no effect on the mRNA expression of proinflammatory or anti-inflammatory cytokines (Park et al., 2013). The intracerebroventricular infusion of corticotrophin-releasing hormone, which is up-regulated in the hypothalamus in mouse models of depression, to wild-type mice also induces anxiety-like behavior and alters colonic function and fecal microbiota profiles (Park et al., 2013). The authors propose that stress-related alterations in the colonic motility may explain the changes in the microbiota profiles.

### Microbiota Disturbance and Host Inflammation are Involved in Behavior Alteration

Although, alterations in the microbiota community are widely reported in those with psychiatric disorders, whether microbiota is a casual factor in regulating neurophysiological behavior is not clear. A recent study in obesity suggests that microbiota tends to be a causal factor in regulating obesity-associated neuropsychiatric disorders (Bruce-Keller et al., 2015). Obesity is linked to an increased risk of neuropsychiatric disorders, such as depression, dementia, and brain pathology (Bruce-Keller et al., 2009). The transplantation of the gut microbiota from HFD-fed mice to recipient mice (microbiota depletion by antibiotics) disrupts exploratory, cognitive, and stereotypical behavior compared to mice given microbiota from a control diet (Bruce-Keller et al., 2015). Moreover, HFD microbiota down-regulates the protein expression of occludin in the jejunum and colon, up-regulates inflammatory markers in the colon, increases plasma endotoxin and neuroinflammation, and disrupts cerebrovascular homeostasis (Bruce-Keller et al., 2015). Thus, it seems that compared to the jejunum, alterations in the colonic epithelial barrier may have a more detrimental effect on inflammation. It also establishes a robust link between gut dysbiosis and neurological dysfunction, and indicates the role of microbiota as a brain peacekeeper.

Other studies on the infection of enteric pathogens also suggest a potential relationship between gut dysbiosis and neurological dysfunction. Pathogen infection induces anxiety-like behavior and changes in the composition of the gut microbiota. Anxiety-like behavior increases in mice infected with *Trichuris muris* (Bercik et al., 2010), *C. rodentium* (Lyte et al., 2006), and *Campylobacter jejuni* (Goehler et al., 2008), respectively. *C. rodentium* infection increases the abundance of Enterobacteriaceae in the colon of mice (Lupp et al., 2007). *C. rodentium* infection also leads to stress-induced memory dysfunction in mice (Gareau et al., 2011). *T. muris* induces anxiety-like behavior via the vagus-independent pathway, whereas the anxiety-related behavior induced by *C. rodentium* seems to be vagus-dependent (Cryan and Dinan, 2012).

In a study of *T. muris* infection, host inflammation, together with the altered microbiota profiles, are found to contribute to the behavioral regulation (Bercik et al., 2010). During *T. muris* infection, etanercept treatment normalizes the behavior and reduces the plasma level of proinflammatory cytokines (tumor necrosis factor α), while does not influence hippocampal *Bdnf* mRNA expression. Interestingly, the administration of probiotic *Bifidobacterium longum* normalizes the behavior and *Bdnf* mRNA expression, while does not affect the concentration of plasma cytokines (Bercik et al., 2010). Therefore, both host inflammation and probiotic function are involved in behavioral regulation. These results hint that the combined therapy, with the host and microbiota treated simultaneously, may be a possible approach to normalize behavior and brain function in those with psychiatric disorders.

### Restoring the Gut Microbiota Balance Benefits Normal Brain Function

The use of probiotic and antibiotic to restore the gut microbiota balance can be an effective strategy to regulate anxiety and stress response. As reviewed previously, probiotics treatment reduces anxiety, decreases the stress response, and normalizes behavior, in both humans and rodents (Cryan and Dinan, 2012; Luna and Foster, 2015). For example, treatment with *L. rhamnosus* reduces stress-induced anxiety- and depression-related behavior via the vagus-dependent pathway, with the improvement related to increased mRNA expression of gamma-aminobutyric acid (GABA)_Aα2_ and decreased expression of GABA_Aα1_ in the hippocampus (Bravo et al., 2011). Another study shows that treatment with *Blautia coccoides* alone reduces the anxiety level in gnobiotic mice, whereas *B. infantis* has little effect on the anxiety level (Nishino et al., 2013). During *T. muris* (Bercik et al., 2010) and *C. rodentium* (Mackos et al., 2013) infection, a probiotic treatment alleviates anxiety-like behavior and normalizes the expression of hippocampal BDNF. Human studies show that the treatment of healthy women with a probiotics mix of *Bifidobacterium animalis* subsp. *lactis*, *Streptococcus thermophiles*, *Lactobacillus bulgaricus*, and *Lactococcus lactis* subsp. *lactis* is associated with changes in midbrain connectivity during an emotional attention task (Tillisch et al., 2013). Specifically, the administration of *L. rhamnosus* + *L. helveticus* before and during *C. rodentium* infection prevents memory dysfunction in mice (Gareau et al., 2011). Thus, these findings confirm the existence of the microbiota-gut–brain axis. The results establish the usefulness of probiotics as new medications against anxiety.

The antibiotic treatment is another approach that modulates the gut microbiota and brain function. Oral antimicrobials increase exploratory behavior and the hippocampal expression of *Bdnf* in SPF mice (Bercik et al., 2011). At the phylotype level, antimicrobials increase the abundance of Firmicutes and Actinobacteria and decrease γ-Proteobacteria and Bacteroidetes in the colon (Bercik et al., 2011). Interestingly, antimicrobials do not affect neurotransmitters and inflammatory cytokines in the gut (Bercik et al., 2011). A potential mechanism may be that changes in the microbiota or microbial products may be involved in the increased hippocampal expression of *Bdnf*. During water avoidance stress, antibiotics administration increases the quantity of *Lactobacillus* and decreases *Clostridium coccoides* cluster XIVa, with the two bacterial groups showing positive and negative correlations, respectively, with the expression of cannabinoid receptor type 2 (Aguilera et al., 2013). This finding points to the modulation of the intestinal endocannabinoid system by gut microbiota. Intracolonic stress induced by capsaicin provokes visceral pain-related responses, which are absent with antibiotic treatment in mice (Aguilera et al., 2013). Therefore, gut microbiota is involved in regulating stress-induced visceral hypersensitivity.

In a rat model using chronic water avoidance or repeat restraint stressors, rifaximin administration increases the expression of the tight junction protein occludin and decreases the expression of proinflammatory interleukin 17, interleukin 6, and tumor necrosis factor α mRNA in the distal ileum, which alleviate visceral hyperalgesia (Xu et al., 2014). The effect of rifaximin is associated with increased abundance of *Lactobacillus* in the ileum. Some *Lactobacillus* species, such as *L. casei*, exert an anti-inflammatory effect in intestinal mucosa (Llopis et al., 2009). Collectively, these findings suggest that the use of probiotics may help to regulate behaviors.

Modulation of the gut microbiota with certain microbe may be adopted to treat ASDs. Some individuals with ASD have comorbid gastrointestinal dysfunction (Coury et al., 2012). In a mouse model of ASD, *Bacteroides fragilis* colonization ameliorates abnormal communicative, stereotypical, and anxiety-like behaviors in maternal immune activation offspring mice (Hsiao et al., 2013). It also improves the integrity of the gut barrier, down-regulates the proinflammatory response, and restores the gut microbiota in the colon (Hsiao et al., 2013). The amelioration induced by bacterial colonization is strain dependent, as no such amelioration is observed following the administration of *Enterococcus faecalis*. The administration of a microbial product 4-ethylphenylsulfate, which increases in response to poly (I:C) activation and seems to induce anxiety-like behavior in wild-type mice, after *B. fragilis* administration may explain the observed improvements following *B. fragilis* colonization (Hsiao et al., 2013). The aforementioned study points to microbiota-mediated regulation of ASD and related gastrointestinal dysfunction.

In summary, the literatures show the importance of the microbiota–gut–brain axis in regulating brain function. The microbiota–gut–brain connection further provides an opportunity for microbiota manipulation to treat neurodevelopmental disorders. Importantly, the findings that restoring the gut microbiota balance benefits normal brain function further support our idea that the gut microbiota is an important brain peacekeeper.

## Circadian Rhythms and Gut Microbiota: Bidirectional Regulation

An increasing number of studies indicate the relationship between circadian rhythms and gut microbiota. The physiological circadian rhythm regulates daily events, including feeding, hormone secretion, and metabolic homeostasis (Liang et al., 2015). The physiological condition can change in response to the oscillation of light during a 24-h cycle. Intestinal functions, such as nutrient absorption and motility, are regulated in a circadian manner (Hussain and Pan, 2009). In particular, food entrainment may link the circadian rhythms of the intestine with the dorsomedial hypothalamus, providing a potential means of the gut–brain communication (Hussain and Pan, 2009).

Alterations in dietary habitats and circadian rhythms may potentially affect the composition of the microbiota community, consequently affecting the host’s metabolism (Thaiss et al., 2014; Liang et al., 2015).

The gut microbiota shows cyclical fluctuations in response to changes in diets or feeding patterns. For instance, the HFD changes diurnal patterns of the gut microbiota in mice (Leone et al., 2015). The HFD also impairs the expression of central and hepatic circadian clock gene in GF mice (Leone et al., 2015). In this case, short-chain fatty acids derived from the microbial metabolism can modulate the expression of circadian clock genes in hepatocytes (Leone et al., 2015). Except for diets, feeding patterns may also change the microbial communities. Time-restricted feeding restores the cyclical fluctuation of the gut microbiota, which is diminished by HFD-induced obesity (Zarrinpar et al., 2014). The restoration of the gut microbiota is accompanied by an increase in the concentration of galactose, deoxycholate, and taurocholate in feces (Zarrinpar et al., 2014).

Interestingly, the host circadian clock in mice regulates microbial circadian rhythmicity (Liang et al., 2015). Bacteroidetes is more predominant during the daytime than at evening time, and variations in the level of Bacteroidetes during the day are the main driving force of circadian oscillations in the total bacterial load (Liang et al., 2015). The deletion of *Bmal1*, one of the core components of the mammalian clock, abolishes the rhythmicity in the fecal microbiota composition, especially in female mice (Liang et al., 2015). Similarly, a deficiency of clock gene *Per1/2* or induction of jet lag in mice leads to aberrant microbiota diurnal fluctuations and dysbiosis (Thaiss et al., 2014). The jet-leg-induced microbiota dysbiosis promotes glucose intolerance and obesity in both humans and mice (Thaiss et al., 2014). The metabolic phenotype produced by jet-leg can be transferred to GF mice by fecal transplantation (Thaiss et al., 2014), which suggests the microbiota-dependent alteration in individuals with abnormal circadian rhythms. This work also highlights the role of diurnal variations in the microbial composition and function in driving metabolic diseases.

The above findings indicate the importance of the host circadian clock in regulating the composition of the gut microbiota assembly. What’s more, the gut microbiota may be a causal factor in regulating the components of circadian clock and the host metabolism.

The ability of the gut microbiota to affect the circadian rhythm is also demonstrated in the studies involved in the antibiotic treatment. Microbiota depletion by antibiotics treatment is associated with deficiencies in the expression of clock genes. For example, microbiota depletion by antibiotics disrupts the oscillatory profile of the transcript expression of TLRs (*Tlr1-5*, *Tlr9*), TLR adaptors (interleukin-1 receptor-associated kinase 4), and inflammatory cytokines (*Il-6*, *Il-1β*, and regenerating islet-derived 3 gamma) throughout the circadian cycle in ileal intestinal epithelial cells (Mukherji et al., 2013). The rhythmic TLR expression mediates microbiota cues to the downstream activators, the c-Jun N-terminal kinase and the inhibitor of nuclear factor-kappa B kinase β. The administration of LPS following microbiota depletion restores the expression of TLRs and clock components (Mukherji et al., 2013). These results point to the essential role of the gut microbiota or microbial cues in regulating the circadian clock. The circadian signals between microbiota and host mucosa provide insight into clock-controlled genes which regulate intestinal immune homeostasis. These findings also expand our understanding of the role of the gut microbiota as a brain peacekeeper.

It becomes clear that there is a bidirectional regulation between circadian rhythm and gut microbiota. However, in case of psychiatric disorders, whether anxiety and ASDs affect the rhythmic oscillations of the gut microbiota, and consequently change host health, is an intriguing question to be further studied.

## Epigenetic Regulation of Neuronal Cell Function

Epigenetic regulation is involved in the regulation of the nervous system (for a review, see Stilling et al., 2014). MicroRNAs exert the epigenetic regulation by post-transcriptionally regulating gene expression of the target mRNAs (Liu and Xu, 2011). They are known to regulate the immune response, epithelial differentiation, CNS trauma, and degenerative disorders (Biton et al., 2011; Liu and Xu, 2011). In neurodegenerative diseases, microRNAs also regulate the survival of neuronal cells, such as Mir433 in Parkinson’s disease and Mir9 in Huntington’s disease (Packer et al., 2008). The presence of the gut microbiota induces the expression of Mir145 in the murine cecum (Singh et al., 2012). Mir145 is important in neural crest function (Strobl-Mazzulla et al., 2012). However, the direct role of the gut microbiota in regulating microRNAs in the nervous system is unclear.

It is possible that the microbial product will affect the components of nervous system. The microbial product butyrate can induce the expression of Mir375 in differentiated human embryonic stem cells (Tzur et al., 2008). The induction of Mir375 promotes colonic goblet-cell maturation during *T. muris* infection in wild-type mice (Biton et al., 2011). Butyrate appears to regulate the number of choline acetyltransferase-immunoreactive myenteric neurons, possibly acting as a histone deacetylase inhibitor (Soret et al., 2010). The expression of histone deacetylase 3 is essential for the barrier function of intestinal epithelial cells, paneth cells development, and maintaining a balanced microbiota (Alenghat et al., 2013). Thus, the epigenetic regulation by butyrate may link the function of the gut microbiota to the nervous system.

A recent study on HFD indicate the role of saturated free fatty acid in regulating the function of enteric neuron. In a mice model, palmitate-induced apoptosis of enteric neurons, and the apoptotic function of palmitate are mediated by HFD-induced up-regulation of Mir375 (Nezami et al., 2014). As mentioned earlier in this review, the dysbiosis of the gut microbiota induced by HFD is associated with obesity-associated neuropsychiatric disorders (Bruce-Keller et al., 2015). Therefore, whether a link exists between the gut microbiota, microRNA, and neural regulation may be worthy of further investigation.

## The Host-Microbiota Interaction May be a Key Process in Mediating the Microbiota–Gut–Brain Axis

The mechanisms by which the gut microbiota regulates the brain health have been discussed previously (Cryan and Dinan, 2012). They include pathways mediated by metabolites, the immune system, and the vagus nerve (Cryan and Dinan, 2012). Based on the literatures discussed above, we propose roles for a pattern recognition receptor (PRR)-mediated interaction and a microbial metabolite-mediated interaction in the microbial regulation of brain health.

### Pattern Recognition Receptor (PRR)-Mediated Interaction: The Microbiota-Associated Molecular Pattern MAMP/PRR-Gut-Brain Hypothesis

Pattern recognition receptors may be involved in mediating the regulation of the gut microbiota. Microbiota-associated molecular pattern (MAMP) and PRRs crosstalk is an essential mechanism for host recognition of microbiota (Mu et al., 2015). It is interesting to note that the intestinal bacteria, such as *Lactobacillus* (Bravo et al., 2011), *Bifidobacterium* (Bercik et al., 2010) and *Blautia* (Nishino et al., 2013), which are used as probiotics to alleviate anxiety behavior, are gram-positive. It is also found that stress exposure usually reduces the abundance of *Lactobacillus* species (Galley and Bailey, 2014). Interestingly, the bacteria enriched after antibiotic treatment are also gram-positive, such as *Lactobacillus* (Llopis et al., 2009; Aguilera et al., 2013; Xu et al., 2014). Such bacteria are known to produce the lipoteichoic acid, a ligand of TLR2.

As shown by studies of *Tlr2*-deficient mice, TLR2 is important in linking the epithelium function of the intestinal microbiota and the ENS (Brun et al., 2013). TLR2 is expressed in enteric neurons, glia, and smooth muscle in the ileum of C57BL/6J mice (Brun et al., 2013). *Tlr2* deficiency leads to the abnormal architecture and neurochemical profile of the ENS, the intestinal dysmotility, and the decreased levels of GDNF in smooth muscle cells, as compared to wild-type mice (Brun et al., 2013). This finding provides an important hint that the integrity of the ENS depends on the microbiota-TLR2-GDNF axis. Furthermore, the dysfunction of the ENS increases the sensitivity to chemical-induced colitis (Brun et al., 2013). As reported earlier, the microbiota dysbiosis in Crohn’s disease patients may affect the normal structure of the ENS, thereby contributing to the spread and exacerbation of the disease (Lakhan and Kirchgessner, 2010).

Based on the above results, we propose that the interaction between lipoteichoic acid and TLR2 interaction may be involved in mediating the microbiota–gut–brain axis. This hypothesis is supported by a recent study demonstrating that lipoteichoic acid treatment down-regulates the expression of phospho-protein kinase B (PKB/AKT) and phospho- glycogen synthase kinase-3α/β, which are up-regulated in a *Tlr2*-deficient mouse model of schizophrenia (Park et al., 2015).

### Microbial Metabolite-Mediated Regulation

Bacteria which are capable of producing short-chain fatty acids are undoubtedly regulators of appetite. Meanwhile, microbiota-mediated tryptophan metabolism also regulates neural function (Yano et al., 2015). The human gut bacteria *Clostridium sporogenes* and *Ruminococcus gnavus* are capable of decarboxylating tryptophan to tryptamine, a β-arylamine neurotransmitter (Williams et al., 2014). Tryptamine induces the release of serotonin by enterochromaffin cells (Takaki et al., 1985). Stress can increase the availability of serotonin in the colon (Julio-Pieper et al., 2012; Aguilera et al., 2013). As shown in a recent study, spore-forming bacteria from mice and human gut microbiota promote colonic serotonin biosynthesis, thus regulating gastrointestinal motility and platelet function (Yano et al., 2015). These literatures indicate the potential mechanism of microbiota-mediated regulation of brain serotonergic system.

Other microbial metabolites, such as phytoestrogens, may also bridge gut and brain functions. Equol is an estrogen produced by the metabolism of dietary daidzein (a kind of soy isoflavone) by some members of the gut microbiota in mammals (Han et al., 2006; Shor et al., 2012). Equol-producing bacteria belonging to the *Eubacterium* have been isolated from porcine feces (Yu et al., 2008). Equol administration exerts neuroprotection against cerebral ischemia/reperfusion injury in rats by decreasing brain histological damage and inhibiting phospho-Src (Yu et al., 2014). The intake of isoflavones has been found to improve memory performance and cognitive behavior (Kennedy, 2014), although the mechanisms remain unclear. Therefore, these findings raise the intriguing idea that gut-derived equol might affect the function of the nervous system.

## Perspectives and Conclusion

The understanding of the role of the gut microbiota in regulating brain health is increasing. Studies of rodent models have expanded the concept of the microbiota–gut–brain axis. However, the community and functions of the gut microbiota are different in rodents and humans (Nguyen et al., 2015). Thus, the findings of rodent research cannot be automatically translated to humans. Even in humans, the microbial community varies between different individuals. The difference in the composition of the gut microbiota may influence the susceptibility of individuals to illness (Aziz et al., 2013). Even though, studies of rodent models can help to shed light on the potential existence of a microbiota–gut–brain axis in humans.

**FIGURE 2:**
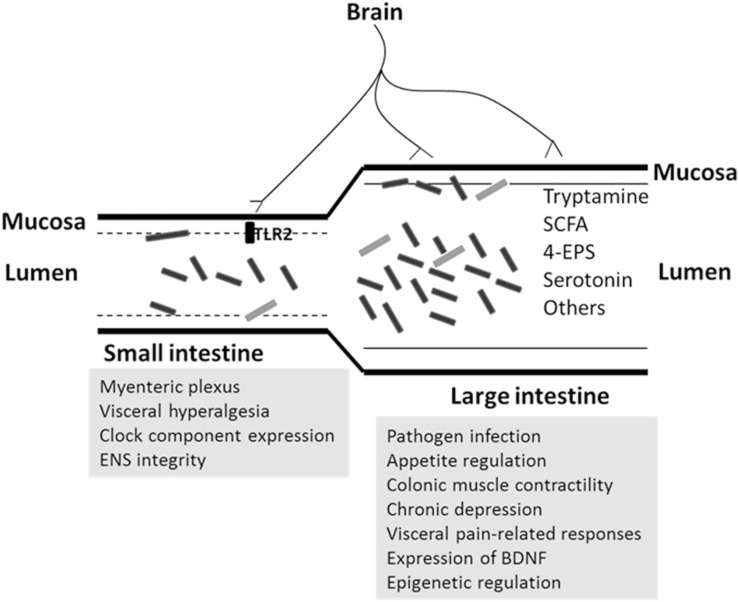
**Potential regulation of microbiota on brain function in small and large intestine.** The microbiota composition differs between small and large intestine, or mucosa and lumen. The rectangle objects are represented as bacteria. The site-related pathways (in gray background) are proposed according to the related discovery in this review.

As mentioned earlier, the spatial distribution of the microbiota in the small and large intestine and in the lumen and mucosa is different (**Figure 2**). Different sites in the gut form a niche-specific environment that differ in pH, oxygen availability, bacterial density, metabolite composition, and other aspects. It is not clear whether the microbiota in the small and large intestine and those in the lumen and mucosa contribute equally to the microbiota–gut–brain axis. Resolving this issue is essential in order to employ appropriate interventions, as the targeted sites of various treatments differ. The absolute microbiota population should also be quantified to define the potential contribution of communities at different sites to the gut–brain axis.

Actually, many studies of human psychiatric disorders use fecal microbiota as an indicator. In such studies, the sample accessibility and ethnicity are major concerns. Generally, changes in the composition of the gut microbiota are usually associated with alterations in microbial gene expression, proteins, or metabolites. These alterations make it difficult to identify the key factors regulating the microbiota–gut–brain axis, especially in studies using fecal analysis. A combined pipeline integrating metagenomics and metabolomics may aid the discovery of important changes beyond those in bacterial numbers.

Further, work is also needed to ascertain the possible contribution of the gut microbiota to brain health. It will be necessary to determine whether abnormal brain function is dependent or independent of the gut microbiota. The existence of microbiota dysbiosis in patients with psychiatric disorders and the relationship between dysbiosis and hyperactivity of the hypothalamic–pituitary–adrenal axis are other areas worthy of further research. According to earlier work, both inflammation-dependent and inflammation-independent pathways are involved in improving brain health (Bercik et al., 2010). The microbiota may function alone or in synergy with other factors, such as inflammation, in regulating brain function. It is also essential to determine whether changes in the composition of the gut microbiota are the cause or the result of certain behaviors. The latter can be elucidated by manipulating the composition and number of the gut microbiota and examining the subsequent effects on brain health.

Obviously, a healthy microbiota community is necessary to maintain a healthy nervous system. Increasing evidences support a peacekeeper role for the gut microbiota in regulating the brain function, due to that the gut microbiota regulates nervous system development, stress responses, anxiety, appetite, and circadian rhythms. The depletion or disturbance of microbial community is associated with psychiatric diseases. Treatments aimed at restoring the normal gut microbiota and intestinal homeostasis are associated with ameliorated neural responses. In summary, identifying the alteration of the gut microbiota can provide a clinical indicator and aid the diagnosis of patients with psychiatric comorbidity.

## Author Contributions

WZ, CM, and YY wrote the paper; CM, YY, and WZ contributed the conception of the work; CM, YY, and WZ revised it critically for important content; WZ had primary responsibility for the final content.

## Conflict of Interest Statement

The authors declare that the research was conducted in the absence of any commercial or financial relationships that could be construed as a potential conflict of interest.
